# Recent Advance in the Sample Pretreatments for Drug Analysis in Zebrafish

**DOI:** 10.3390/ph19030465

**Published:** 2026-03-12

**Authors:** Ting Wang, Chuyu Wang, Mingjing Luo, Xinyu Wang, Yiwen Chen, Zhi Yang, Guang Hu, Weikang Liu

**Affiliations:** 1 School of Pharmacy and Bioengineering, Chongqing University of Technology, Chongqing 400054, China; ting@stu.cqut.edu.cn (T.W.); luomj@stu.cqut.edu.cn (M.L.); dayu_0512@stu.cqut.edu.cn (X.W.); 2860210189@stu.cqut.edu.cn (Y.C.); 1772129869@stu.cqut.edu.cn (Z.Y.); 2 School of New Materials and Chemical Engineering, Beijing Institute of Petrochemical Technology, Beijing 102627, China; 2024310261@bipt.edu.cn; 3 Department of Hepatobiliary and Pancreatic Surgery, Peking University First Hospital, Beijing 100034, China

**Keywords:** zebrafish, sample pretreatment, solid phase extraction, liquid phase extraction

## Abstract

Zebrafish, as an emerging model organism, are widely used in in vivo pharmacodynamic and pharmacokinetic studies. Unlike direct chemical analyses that require no sample preparation, most biological samples must undergo preprocessing steps—procedures that profoundly affect analytical outcomes. This paper systematically summarizes the main methods and types of zebrafish sample pretreatment currently in use, aiming to provide a reference for future research in zebrafish sample analysis and preparation. A systematic search was conducted across PubMed, Web of Science, and CNKI for studies published between 2014 and 2024 focusing on liquid–liquid extraction (LLE), solid phase extraction (SPE), and related techniques for zebrafish drug analysis. The results indicate that traditional methods, including LLE and protein precipitation, remain prevalent due to their operational simplicity, but are limited by low enrichment efficiency and pronounced matrix effects (MEs). In contrast, advanced SPE techniques, particularly solid phase microextraction (SPME), are increasingly favored for complex biological sample processing, with key trends including technique hyphenation (e.g., SPME–high-performance liquid chromatography (SPME-HPLC), and micro-SPE–mass spectrometry (µSPE-MS)) and the development of novel sorbents. Despite these advances, current challenges persist, such as immature rapid on-site pretreatment protocols, the difficulty of balancing analytical efficiency with operational simplicity, and the lack of standardized procedures across studies. Overall, zebrafish sample pretreatment techniques are evolving toward higher efficiency, selectivity, and automation. Future research efforts should prioritize the development of intelligent, eco-friendly pretreatment methods and the establishment of unified standards to enhance the reproducibility and comparability of zebrafish-based pharmacological studies.

## 1. Introduction

An accurate assessment of the efficacy and safety of drugs is critical through the drug development process. The zebrafish (Danio rerio), a small tropical freshwater species, has become an extensively utilized model in biological studies due to its rapid development, high reproductive capacity, and the conservation of numerous human genes [1,2]. The transparency of zebrafish embryos throughout early developmental stages enables non-invasive imaging of internal organs and physiological processes, making it an ideal organism for research in developmental biology, toxicology, and disease mechanisms [3]. In terms of drug analysis, zebrafish offer several advantages, including their ability to recapitulate human metabolic pathways [4]. However, substantial species-specific differences exist, particularly in cytochrome P450 enzyme activities and phase II conjugation pathways, which may result in divergent drug metabolic rates and metabolite profiles [5].These metabolic differences have direct implications for the design of sample pretreatment strategies. For instance, higher metabolic rates may require shorter sampling intervals to ensure reliable detection of parent compounds before extensive biotransformation [6]. Furthermore, the occurrence of unique or abundant metabolites may demand customized extraction and cleanup procedures. In addition, interspecies variations in protein-binding affinity can affect analyte recovery during LLE and SPE, as compounds with strong binding affinity may exhibit poor partitioning into organic solvents or low adsorption onto sorbents. These metabolic characteristics, combined with heightened sensitivity to a wide range of drugs and toxins, and relatively low cost and ease of maintenance when compared to other animal models, make zebrafish a valuable platform for pharmaceutical research [7,8,9,10]. Notably, the zebrafish model provides a highly efficient, rapid, and convenient approach for evaluating the biological activity, metabolic pathways, and potential toxicity of pharmaceuticals [11,12,13]. In studies assessing the effects of plant-derived compounds, researchers typically dissolve them in the zebrafish’s culture water to create a preset concentration gradient [10,14,15]. Following exposure, zebrafish are collected and subjected to sample pretreatment procedures (e.g., homogenization, extraction, purification) before analysis by high-performance liquid chromatography (HPLC), liquid chromatography–mass spectrometry (LC-MS), or other techniques [14,16,17,18].

However, analyzing pharmaceuticals in zebra fish samples presents several challenges. The zebrafish biological matrix, which contains proteins, lipids, and other macromolecules, may interfere with analytical methods, potentially resulting in inaccurate results [7]. During sample pretreatment, these constituents may be co-extracted with target analytes and cause interference in instrumental analysis, especially in LC-MS/MS-based detection [19,20]. Such MEs usually lead to ion suppression or enhancement, thereby reducing quantification accuracy and sensitivity [21,22]. Conventional extraction approaches such as LLE and SPE frequently suffer from notable MEs, with ion suppression ranging from 20% to 35% [23,24]. To mitigate MEs, various strategies have been established, including selective extraction techniques (e.g., SPME, magnetic solid phase extraction (MSPE), dispersive solid phase extraction (DSPE)), matrix-matched calibration, internal standardization using isotopically labeled compounds, and optimized chromatographic separation. Furthermore, the concentrations of medicines and their metabolites in zebrafish samples are often low, requiring the use of highly sensitive and specific analytical techniques [18]. Consequently, sample pretreatment becomes a critical step in ensuring the success of drug analysis in zebrafish. Sample pretreatment involves a series of procedures designed to extract, purify, and concentrate target analytes from the biological matrix. These processes are critical for reducing matrix interference, enhancing analyte detection, and improving the overall quality of analytical data [25].

Recent advances in sample pretreatment techniques for drug analysis in zebrafish have focused on improving their efficiency, selectivity, and automation [26,27]. For precise quantitative analysis, it is essential to employ an appropriate extraction medium, a rational extraction method, and suitable auxiliary enhancement technologies during the pretreatment phase to optimize extraction efficiency. Compared to traditional extraction techniques such as centrifugation and filtration, novel extraction methods like LLE and SPE offer enhanced operational convenience and improved recovery rates, making them increasingly popular in zebrafish drug analysis [22,28]. LLE is fundamentally an equilibrium process governed by the Nernst distribution law, primarily determined by the logarithmic partition coefficient (log P) of analytes [29]. The log P value reflects a compound’s relative hydrophobicity: Analytes with high log P values tend to partition into non-polar organic solvents, whereas those with low log P values remain predominantly in the aqueous phase [30]. Besides log P, extraction efficiency is influenced by analyte–matrix interactions [31]. For example, the binding of analytes to proteins or lipids in zebrafish homogenates reduces free analyte concentration and extractability, which can be mitigated by protein denaturants or pH adjustment to enhance target recovery.

In contrast, SPE uses solid sorbents to selectively retain target compounds via hydrophobic interactions, electrostatic attraction, and hydrogen bonding [32]. The adsorption process is typically described by equilibrium models (e.g., Langmuir and Freundlich isotherms), which characterize analyte distribution between the sample solution and sorbent surface [33]. In complex matrices such as zebrafish homogenates, thermodynamic factors (e.g., competitive adsorption between target analytes and co-extracted matrix components, or matrix-induced changes in activity coefficients) impair recovery [34]. These interferences can be minimized by using highly selective sorbents or incorporating additional cleanup steps into the extraction protocol. Recently, advancements have been made in SPME and liquid phase microextraction (LPME), building upon the principles of SPE and LLE, respectively. Microextraction methods, such as hollow fiber liquid phase microextraction, offer several advantages, including reduced solvent consumption, simplified procedures, and higher enrichment factors [35]. Supercritical fluid extraction, on the other hand, utilizes carbon dioxide or other supercritical gases under high-pressure and high-temperature conditions to extract analytes, offering improved selectivity and reduced environmental impact [36].

The unique biological characteristics of zebrafish directly influence the design of sample pretreatment protocols. Their small size necessitates the use of whole-body homogenates, which are complex matrices rich in lipids, proteins, and pigments [37,38]. These endogenous components frequently interfere with both analyte extraction and instrumental detection. The metabolic divergences noted above further compound these challenges by shaping the analytical target. Specifically, differences in enzyme activities determine whether the parent drug or its metabolites are the analytes of interest, which in turn dictates the choice of extraction conditions—for instance, necessitating hydrolysis steps to release conjugated metabolites. Importantly, conventional sample preparation methods optimized for plasma, cell cultures, or other fish species cannot be directly applied to zebrafish matrices without rigorous re-optimization [39,40]. This underscores the urgent need for tailored extraction strategies. In response, increasing efforts have focused on customizing techniques specifically for zebrafish homogenates, including the selection of appropriate sorbents, the optimization of solvent systems, and the refinement of cleanup procedures to address matrix-specific challenges.

This review comprehensively evaluates studies published from 2014 to 2024, and introduces the sample pretreatment techniques for zebrafish pharmaceutical analysis based on LLE and SPE and their related extraction methods, as well as their applications in real sample matrices. Zebrafish research encompasses different developmental stages (embryos, larvae, and adults), each imposing distinct demands on sample pretreatment. Embryos and larvae provide minimal sample amounts (typically <1 mg protein per individual) and high water content, necessitating miniaturized extraction protocols and highly sensitive detection. Adult zebrafish, in contrast, contain higher lipid and protein levels, which increase matrix complexity and require more rigorous cleanup procedures. Although most studies cited in this review involve adult zebrafish, the techniques described can be readily adapted to early life stages through simple adjustments, such as reduced solvent volumes or sample pooling, as the fundamental principles of extraction remain consistent across all homogenized samples. This review specifically highlights zebrafish biospecimens as target analytes, explores a variety of sample pretreatment strategies, and provides detailed analysis data of related technologies to assist researchers in selecting optimal sample pretreatment methods [41].

## 2. Zebrafish Samples Underwent Liquid Phase Extraction

### 2.1. Liquid Phase Extraction Form

For zebrafish analysis, an extraction process is required to extract the analyte of interest. Zebrafish biological samples typically exhibit a complex multi-component matrix, posing challenges for separation and analytical equipment, particularly in liquid chromatography (LC). Therefore, researchers have shown great interest in traditional liquid–liquid extraction, accelerated solvent extraction, hollow fiber microextraction and liquid–liquid microextraction.

LLE is one of the simplest, fastest, and most economical methods among various pretreatment techniques [42]. Furthermore, the substances of interest in zebrafish biological samples are often present at low concentrations, necessitating additional concentration steps for subsequent detection. Consequently, emerging sample pretreatment techniques such as supercritical fluid liquid–liquid extraction (SFLLE), accelerated solvent extraction (ASE), hollow fiber liquid–liquid microextraction (HF-LLME), and dispersive liquid–liquid microextraction (DLLME) have gained widespread acceptance, as shown in Table 1. Table 2 summarizes the key LLE and solvent systems discussed below, along with their recommended applications and limitations.

#### 2.1.1. Traditional Liquid–Liquid Extraction

Traditional LLE is performed by using sample components that have different solubility in different solvents for separation. This extraction process transfers solutes from one solvent to another. Traditional liquid–liquid extraction is divided into three steps: combining the sample with the solvent, separating, and recovering the needed solvent [59]. Lipid-rich zebrafish samples frequently suffer from severe emulsion formation during the separation step, which greatly complicates LLE [60]. Endogenous lipids and phospholipids can act as natural surfactants, stabilizing finely dispersed solvent droplets and forming a persistent emulsion at the liquid–liquid interface. This issue impedes or even prevents complete phase separation, sequestering a fraction of target analytes within the emulsion layer and consequently compromising extraction recovery and reproducibility. To alleviate emulsification in lipid-rich zebrafish homogenates, multiple strategies can be adopted, including increasing centrifugal force and time to facilitate droplet coalescence, incorporating demulsifying agents such as salts or short-chain alcohols to disrupt the interfacial film, and adjusting solvent systems to reduce interfacial tension [47]. Resolving these emulsification-related obstacles via optimized separation conditions is critical for obtaining reliable and reproducible results in LLE workflows using zebrafish matrices.

Zebrafish samples were examined in a number of methods following typical liquid–liquid therapy. These research approaches can be broadly classified into biochemical detection and analytical detection.

- Biochemical detection after sample pretreatment:
  1. When it is essential to investigate the effects of plant components on biochemical indicators (triglycerides, total cholesterol, aspartate aminotransferase, alanine aminotransferase, etc.) or zebrafish genes, samples will be subjected to additional biochemical assays [61,62].
  2. When evaluating zebrafish biochemical parameters (triglycerides, total cholesterol), researchers utilized ethanol as an extraction solvent [16,63]. Zebrafish samples were mixed with ethanol (1:9 g/mL) and crushed [64,65]. The mixture was centrifuged to separate the analyte, which was now present in the ethanol layer solution. Following the removal of the upper solution, glycerol-3-phosphate oxidase-peroxidase and other methods were employed to assess the effects of plant components on zebrafish biochemical markers.
  3. When analyzing zebrafish biochemical indicators (aspartate aminotransferase, alanine aminotransferase), researchers employed normal saline as an extraction solvent [66]. Zijin Xu et al. added a 9-fold volume of normal saline to zebrafish samples and mechanically homogenized them in an ice bath [66]. Crocin’s effects on zebrafish AST and ALT can be identified with Reisch colorimetry.
  4. Lo’pez-Serrano Oliver et al. exposed zebrafish to inorganic arsenic and tributyltin, then treated the samples with low RIPA buffer for Western blot analysis [67]. During extraction, the processed samples were placed in an ultrasonic machine and sonicated. This approach speeds up the extraction process while preventing evaporation and dilution of the extraction solvent. Camilla Della Torre and coworkers used a chloroform/methanol/water mixture (4:1:3 *v*/*v*) to precipitate protein in zebrafish for functional proteomics investigation [68].
- Analytical detection after sample pretreatment:

Analytical testing involves using tools to assess the residual of plant chemicals in zebrafish samples after pretreatment. Significant advantages of traditional liquid–liquid extractions include easy operation, controllable experimental parameters, and operation at room temperature [69]. It is worth noting that there are numerous variations in traditional liquid–liquid extraction, in which the purification and enrichment processes or the auxiliary extraction mode can be changed [70].

Traditional liquid–liquid extractions designed for the processing of zebrafish samples are classified into three types based on the purification and enrichment phases: (1) adding surfactants to increase sample fluidity [67]; (2) filtration through the filter membrane to promote the purity of the sample [42,71,72]; and (3) nitrogen was utilized for sample enrichment [51,73].

Most analytical testing tools require liquids with sufficient mobility. Qiang Li et al. from the College of Science at China Agricultural University used 15 to 20 mL of nitric acid and 5% glacial acetic acid to extract inorganic arsenic and tributyltin from zebrafish for graphite furnace atomic absorption spectrometry [67]. The sample solution containing arsenic was too viscous for further investigation. They added 0.04% Triton X-100 surfactant during sample preparation to minimize viscosity and aid in separation from interfering compounds [67]. The limit of detection (LOD) was 1.0 ng/g with a relative standard deviation (RSD) of 13 to 20% and a recovery of >90%.

When using liquid chromatography–mass spectrometry (LC-MS) detection, the material entering the instrument must be particle-free [74]. Xiaohong Yanga et al. extracted pyraoxystrobin using 20 mL of acetonitrile and added 30 mg of zirconium dioxide-coated silica sorbent to the solution to eliminate interfering particles such lipoproteins [71]. Additionally, they used 0.22 μm membrane filtration to eliminate big particle debris prior to injection. The LOD of pyraoxystrobin was 0.01 mg/kg, with RSDs of 0.94–3.57%, a linearity range of 0.01–0.3 mg/kg, and a recovery rate of 98.31–105.61% [71].

Yayuan Li et al. used a mixture of n-hexane and dichloromethane to extract polycyclic aromatic hydrocarbons from zebrafish [51]. After finishing the initial extraction, they concentrated the material to 2 mL with a moderate stream of nitrogen before filtration analysis. Such a concentration step enriches the analyte while preventing thermal deterioration of the sample. The LODs for polycyclic aromatic hydrocarbons were 0.05–0.10 μg/L.

It should also be mentioned that the traditional liquid–liquid extraction procedures used for the pretreatment of zebrafish samples are classified into three types based on the aided extraction mode: (1) traditional liquid–liquid extraction assisted by vortex [75]; (2) traditional liquid–liquid extraction assisted by ultrasound [76]; and (3) traditional liquid–liquid extraction assisted by centrifugation [77].

Stephan Brox et al. proposed a novel methanol-based vortex-assisted traditional liquid–liquid extraction method. Methanol exhibits polar variations and lipid solubility qualities [78]. To prepare the zebrafish samples, methanol was mixed with washed zebrafish embryos. The mixture was be vortexed for 1 min to facilitate the extract caffeine, theophylline, and other chemicals into the aqueous phase, and the resulting sample was identified and analyzed by HPLC. The extraction technique’s linearity was proven in the caffeine concentration range of 1.00–200 ng/mL, with LOD of 0.5 ng/mL. Caffeine recovery was 93–105%.

Xiaotong Du et al. proposed ultrasound-assisted micellar cleanup (UAMC) to process and analyze zebrafish samples with HPLC [45]. They homogenized the zebrafish samples, mixed them with a pretreatment solution comprising TX-114, NaCl, and formic acid, and then placed them in an ultrasonic bath for 8 min. Centrifugation was used to separate the sonicated mixture into two phases: The upper layer was aqueous, while the lower layer was micelle-rich. At this point, the aqueous phase contained the drug to be detected, cefothiazide. The developed technique showed good linearity at cefothiazide concentrations ranging from 0.15 to 489 μg/g. The LOD was 0.038 μg/g, RSDs ranged from 1.3% to 6.4%, and the recovery rates were 97.6–109.7% [45].

Stanislav Kislyuk et al. developed a method for drug analysis with a low sample size (one zebrafish per drug) [79]. To effectively extract nine medications with various physicochemical characteristics (amitriptyline, bupropion, cetirizine, cimetidine, etc.) from zebrafish larvae, they centrifuged the homogenate. This sample preparation method produced modest MEs (81–106%) and high recoveries (74–100%) following ultra-high-performance liquid chromatography–tandem mass spectrometry (UHPLC-MS/MS) analysis.

LLE remains widely used due to its operational simplicity, cost-effectiveness, and adaptability to various detection methods, including both biochemical assays (e.g., triglyceride analysis, aspartate transaminase/alanine transaminase (AST/ALT) measurements) and instrumental techniques such as LC-MS and graphite furnace atomic absorption spectrometry (GFAAS). However, this technique also presents several inherent limitations.

The primary disadvantages of traditional liquid–liquid extraction using various solvents are the huge amount of extraction solvent required and the low extraction efficiency [80]. Such low efficiency can be explained by several mechanistic factors. First, polarity mismatch between target analytes and the extraction solvent results in unfavorable partition behavior, leaving a considerable portion of analytes in the aqueous phase [72]. Second, incomplete mass transfer (especially in viscous zebrafish homogenates) restricts the rate at which analytes diffuse toward and across the liquid–liquid interface, particularly under insufficient mixing conditions [72,81]. Third, analyte–protein binding within the biological matrix can sequester analytes, thereby preventing their release into the organic phase even under thermodynamically favorable partitioning conditions [72]. To overcome these limitations, various advanced techniques such as ASE have been developed to improve extraction efficiency by enhancing mass transfer and disrupting analyte–matrix interactions. The inability to extract in emulsions limits the application of traditional LLE in the analysis of zebrafish samples [82].

#### 2.1.2. Accelerated Solvent Extraction

ASE is a solid or semi-solid sample preparation technique [83]. ASE is primarily based on the application of high pressure in a closed vessel, which translates into high temperature and alters the interaction between solute, solvent, and sample matrix, thus shortening the extraction time [84].

Giuliana Ottonello et al. devised a method for determining polychlorinated biphenyls in zebrafish that combines ASE with gas chromatography–mass spectrometry (GC-MS) [58]. Polychlorinated biphenyls were extracted from the samples using a 33 mL stainless steel extraction cell. The optimal ASE conditions were acetone/n-hexane (1:1, *v*/*v*), a single extraction cycle at 100 °C (5 min heating and 5 min static time), and 10 MPa. Following extraction, the solutes were collected, purified using a solid phase extraction column, and evaluated by GC-MS. The Extrelut-NT3 cartridge contained diatomaceous earth, which served as a solid carrier for fat digestion. Under ideal conditions, LODs ranged from 0.4 to 1.1 ng/g, and limit of quantitations (LOQ) ranged from 1.3 to 3.8 ng/g.

Recently, a modification of the approach of Wardrop et al. [85] has been proposed. After homogenizing zebrafish biosamples, they utilized a Dionex™ ASE™ 350 Accelerated Solvent Extractor (Thermo Fisher Scientific, Waltham, MA, USA) accelerated solvent apparatus to extract 9-nitroanthracene [86]. The use of the ASE method can be a good way to remove the residual surfactant and any existing chemical contaminants in the sample. Thus, the effects of a single pollutant, 9-nitroanthracene, on zebrafish may be investigated.

In ASE, similar to conventional LLE, analyte enrichment is governed by the differential solubility of target compounds in immiscible solvents. However, unlike traditional LLE, ASE is conducted under elevated temperature and pressure, which markedly improves extraction efficiency. Despite this advantage, the high temperatures employed during ASE may lead to the degradation of thermally labile compounds, potentially resulting in underestimated analyte concentrations. Therefore, when applying ASE to thermally sensitive analytes, it is necessary to carefully optimize extraction conditions or consider alternative low-temperature techniques to strike an appropriate balance between extraction performance and compound stability.

#### 2.1.3. Hollow Fiber Liquid–Liquid Microextraction

HF-LPME can operate in two-phase and three-phase modes [35,87]. In the two-phase mode, the hollow fibers must be impregnated in the acceptor phase first. The two-phase mode is mostly employed for analytes with medium-to-low polarity. In the three-phase mode, the immobilized organic solvent is exposed to two aqueous phases: the sample solution and the acceptor phase. The three-phase mode is appropriate for analytes that contain ionizable groups [87].

**Figure 1:**
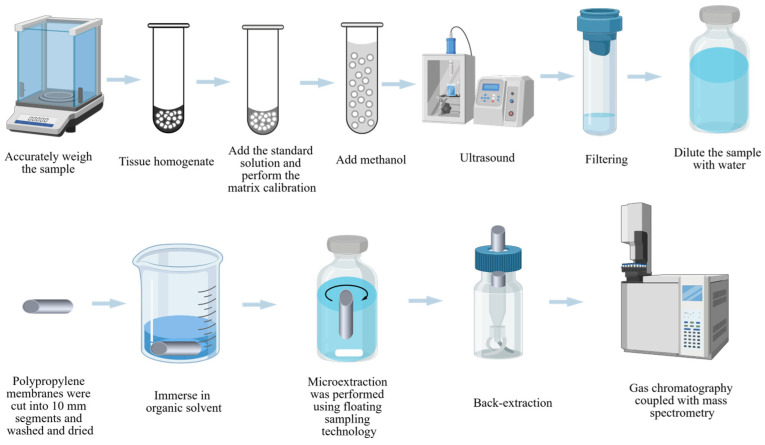
Sample pretreatment process for detecting 18 types of PAHs using HF-LPME and GC-MS.

As shown in Figure 1, Alessandra H. Ide1 et al. devised a method for determining chemicals in zebrafish using HF-LPME in two-phase mode and GC-MS [35,41]. The hollow fibers were initially soaked in toluene for 10 s to saturate their pores. The fibers were then immersed in the sample solution, and the aqueous phase stirring rod was turned on for extraction. Following extraction, the solvent in the fiber lumen was evaluated by GC-MS. Under ideal extraction and derivatization circumstances, this method’s enrichment coefficient ranged from 158 to 279, with an LOD range of 0.017–0.033 g/L (at a signal-to-noise ratio of 3). It is worth mentioning that HF-LLME can be used not only for sample pretreatment of zebrafish, but also for sample pretreatment of canned fish and common fish [87,88].

Meanwhile, it should be noted that the enrichment performance of HF-LPME is highly dependent on the physicochemical properties of the target analytes. Compounds with higher polarity or larger molecular weights generally exhibit lower partition coefficients into the organic membrane phase and slower transmembrane diffusion rates, leading to substantial variations in enrichment factors, even among structurally similar compounds [89]. For instance, Shang Li et al. applied HF-LPME to the analysis of flavonoids with different molecular weights and reported enrichment factors ranging from 9 to 171, corresponding to a 20-fold difference [90]. These findings indicate that, while HF-LPME offers excellent enrichment for non-polar to moderately polar compounds, its applicability to more polar or higher-molecular weight pharmaceuticals necessitates case-specific optimization of extraction parameters, such as the membrane solvent, pH gradient, and extraction time.

HF-LPME exhibits remarkable merits, including high enrichment factors (up to 279-fold in the referenced study), minimal organic solvent consumption, and efficient sample cleanup enabled by the membrane barrier. Its dual-mode operation affords flexibility for the extraction of analytes with diverse physicochemical properties. Nevertheless, this technique is restricted by relatively long extraction durations, susceptibility to air bubble formation, and requirements for manual intervention that may compromise reproducibility. Moreover, the fragility of hollow fibers and the risk of solvent leakage further necessitate meticulous experimental manipulation.

#### 2.1.4. Dispersive Liquid–Liquid Microextraction

DLLME is the most rapidly evolving LLME [91]. The extractant in DLLME will create an emulsion in the sample solution [92]. This increases the contact area between the extractant and the sample, resulting in the enrichment of the target substance into the extractant. The rapid attainment of equilibrium in DLLME is attributed to the exceptionally large interfacial area generated by dispersing the extractant as fine droplets [93]. This configuration significantly shortens the diffusion path of analytes from the aqueous phase into the extractant, enabling equilibrium to be reached within seconds. However, both extraction performance and reproducibility are critically governed by the size and uniformity of the dispersed droplets [92,93]. While smaller droplets enhance the extraction rate by increasing the surface area, they are also more susceptible to coalescence or incomplete phase separation, which can compromise recovery reproducibility. Therefore, precise control over dispersion conditions (the type and volume of disperser solvent, injection parameters, and centrifugation settings) is essential to achieve consistent and reliable results in DLLME. DLLME has the following advantages: efficient enrichment, simplicity of operation, less solvent consumption, and rapid equilibrium establishment [92].

**Figure 2:**
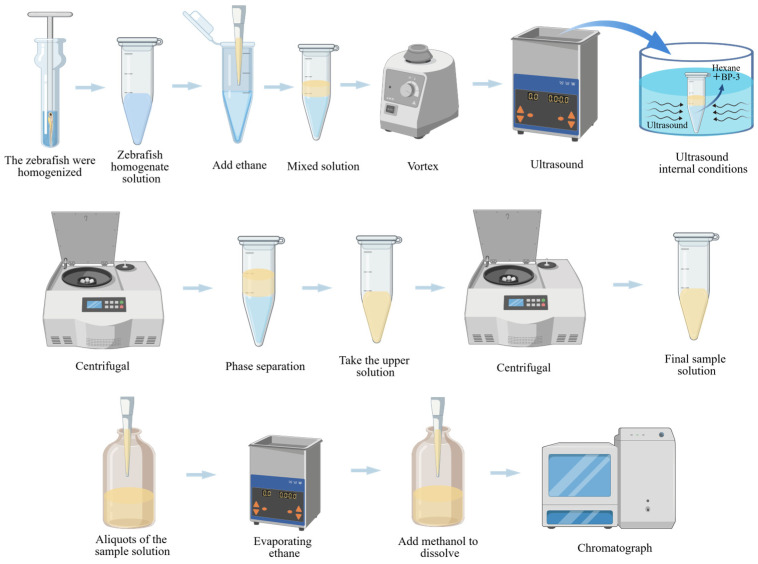
The sample pretreatment process for detecting oxybenzone in zebrafish using DLLME.

Some researchers have proposed the use of ultrasound-assisted DLLME [94]. In this technique, deionized water is added to zebrafish for homogenization [94]. As shown in Figure 2, the homogenized samples were combined with hexane isomer solution and pre-filtered via a 0.45 micronfilter [41]. Finally, the samples were sonicated in an ultrasonic machine for 2 h before being tested using HPLC. The recovery of oxybenzone in zebrafish samples ranged from 98% to 107%, with an LOQ of 0.012 mg/L and an LOD of 0.004 mg/L. Ultrasound-assisted DLLME has distinct advantages. It is a non-thermal extraction process that prevents the destruction of heat-sensitive and heat-labile compounds, preserving the extract’s biological activity.

Although DLLME enables rapid, efficient enrichment with minimal solvent consumption, its performance is highly dependent on the precise control of dispersion conditions and the careful selection of eco-friendly solvents.

### 2.2. Liquid Phase Extraction Solvent

Various extraction solvents were utilized to purify and enrich analytes during zebrafish sample preparation. Conventional organic solvents and mixed solutions are commonly utilized. These extraction solvents are being actively investigated and used. Organic solvents are currently preferred because they suit the pretreatment requirements for zebrafish samples. Organic solutions can selectively extract analytes, limit impurity interference, and increase extraction purity.

#### 2.2.1. Single Organic Solvent

Zebrafish samples include a high concentration of impurities, such as protein. The use of organic solvents can avoid these impurities from entering the subsequent analysis, thereby improving the accuracy and sensitivity of the analysis. Furthermore, zebrafish biosamples contained extremely low concentrations of the target analyte [95]. As a result, multi-stage extraction and purification are required to improve analyte recovery and purity [95]. Organic solvents, due to their particular solubility and selectivity, have become important tools for isolating and purifying target molecules from biological samples. Depending on the nature of the desired chemical, researchers frequently use different organic solvents.

In zebrafish research, the composition of the organic solvent utilized has a significant impact on the extraction efficiency and purity of analytes such as isopropanochlor enantioisomers, fipronil, cyclophosphamide, benzoic acid, and so on [49,54,78]. The solubility of individual analytes varies significantly across organic solvents due to changes in molecular structure and polarity. Polar solvents, including methanol, ethanol, and acetone, are frequently employed to extract polar chemicals from zebrafish samples because of their strong interactions with polar molecules. Non-polar solvents, such as diethyl ether and hexane, are better suited to the extraction of non-polar lipids and metabolites [51,52,96].

#### 2.2.2. Mixed Solution

During the pretreatment of zebrafish samples, the researchers extracted analytes using several combinations. These mixed solutions are often made up of varying quantities of organic solvents or organic solvent–aqueous solution mixes [47,54]. The mixed solution is primarily for some special analytes and will include some salt solutions [68].

The extraction efficiency and selection efficiency of analytes are affected by the properties of the mixed solution. The combination of varied amounts of organic solvents can broaden the solvent system’s coverage and optimize extraction conditions, boosting analyte extraction efficiency [97]. Karyn Le Menach et al. used a single solvent dichloromethane for polycyclic aromatic hydrocarbon (PAH) extraction, while Yayuan Li et al. used a mixed solution of hexane and dichloromethane for PAH extraction [50,51]. The inclusion of n-hexane changes the polarity and solubility of the combined solvent, making it better suited for extracting certain PAHs in zebrafish. The recovery of PAHs was enhanced by 70 to 120% utilizing mixed solution extraction [51]. The inorganic salt sodium chloride may have a salting-out effect, increasing the effectiveness of analyte extraction with an organic solvent like formic acid [45,98]. It is worth mentioning that the salting-out effect was established by the HPLC detection of cefotaxime in zebrafish in a liquid–liquid extraction system using sodium chloride and formic acid as extractants [45].

The preparation of samples is the bottleneck in drug analysis in zebrafish; hence, speed is extremely important. As shown in Table 2, the combined solution has a substantially higher extraction speed than the single solvent. A significant limitation of the mixed solution is its high viscosity, which would pose a greater challenge for subsequent analysis. The use of high viscosity solvents can affect the repeatability of the analytes in the pretreatment process, as well as the standard deviation of the analytical data. As a result, the mixed solution will be diluted with a specific amount of water [47]. People are still working for a better mixing method to improve the effectiveness of analytical data. Recently, Yipo Xiao et al. proposed the use of a mixed solution of methanol, water, and acetamide [47]. In this investigation, the methanol and water mixture had a high surface tension, which allowed the droplets to remain on the sample surface and inhibit diffusion. The addition of acetamide can improve the ionization efficiency of the target analyte in the negative ion mode, increasing signal intensity. The devised analytical detection method was successfully applied to the droplet extraction surface analysis–MS imaging study of perfluorinated octylic acid and perfluorooctane sulfonates in zebrafish [47].

## 3. Zebrafish Samples Underwent Solid Phase Extraction Form

### 3.1. Solid Phase Extraction Form

Zebrafish samples have been pretreated using procedures such as SPE, SPME, DSPE, and MSPE. Table 3 summarizes sample preparation procedures for measuring chemicals in zebrafish samples with instrumental analysis. The table includes sample preparation procedures, adsorbents, and detection techniques. At the same time, this table shows LODs, RSDs, linearity range, and recovery rate for each method. Table 4 provides a comprehensive summary of these techniques and corresponding sorbent materials, along with their recommended applications and zebrafish-specific considerations.

#### 3.1.1. Traditional Solid Phase Extraction

SPE pretreatment of zebrafish samples is based on the principle of selective adsorption and elution. SPE can accomplish the goal of trace or micro analysis by separating and enriching the target analytes in the complex zebrafish sample. Many researchers have used materials with strong affinity for zebrafish samples in order to extract target analytes [108].

Fangfang Chen et al. developed a liquid chromatography–tandem mass spectrometry (LC–MS/MS)-based procedure for trace analysis of 11 drugs (bisphenol A, carbamazepine, diclofenac, fluoxetine, fenofibrate, ibuprofen, naproxen, etc.) in zebrafish [22]. The extraction procedure combines an SPE technology pre-concentration step with ultrasound-assisted extraction. MEs were evaluated using a post-extraction spiking method, and matrix-induced signal variations were compensated for through a multi-internal standard calibration strategy. Specifically, isotopically labeled surrogates with physicochemical properties similar to those of the target analytes (e.g., propranolol-d7) were added to the samples prior to extraction to correct for recovery losses and matrix interferences during sample pretreatment. Additionally, 25 ng of ^13^C_6_-2,4,5-trichlorophenoxyacetic acid was added as an injection internal standard to the final extract before instrumental analysis to account for injection variability and to determine the absolute recovery of the surrogates, thereby ensuring the accuracy and reliability of the quantitative results. Following pretreatment of the samples using this method, the ME of the four medications (diclofenac, gemfibrozil, ibuprofen, and naproxen) was 100%, indicating that the ME was negligible. Within nine minutes, both positive and negative ions were found. For every drug, the LOQs varied from 0.15 to 4.33 pg/µL, and the LODs varied from 0.015 to 0.69 pg/µL [22]. Meanwhile, Yan Zhou et al. developed a highly sensitive ultra-performance liquid chromatography–tandem mass spectrometry (UPLC-MS/MS) method for trace analysis of triptolide in zebrafish embryos [100]. Following treatment with an Oasis™HLB solid phase extraction cartridge, triptolide’s ME varied between 83.3% and 102.5%. Ibuprofen and diclofenac are commonly extracted from human plasma using simple protein precipitation with acetonitrile, achieving recoveries >90% with minimal MEs [109,110]. In zebrafish, however, the higher lipid content necessitates additional cleanup steps, such as the SPE procedures described by Chen et al., to achieve comparable sensitivity. This contrast underscores the need for zebrafish-specific method optimization even for well-characterized analytes.

Chunyan Zhu et al. described a high-throughput screening method based on solid phase extraction technology for rapid screening and micro analysis of the active ingredient of Xiaoke pills in zebrafish [111]. They extracted homogenized zebrafish samples using saline. Waters Oasis SPE cartridges were used in the sample pretreatment program for the purification and concentration of relevant materials. In order to achieve the quick examination of components of traditional Chinese medicine, the final extracted chemicals were analyzed using ultra-high-performance liquid chromatography–high-resolution mass spectrometry (UPLC-HRMS) [111].

SPE is a crucial separation and enrichment method that makes it easier to selectively enrich trace medications in zebrafish. At the same time, SPE combined with LC-MS/MS and UPLC-MS/MS can greatly improve the sensitivity and selectivity of detection, making it an indispensable tool for the analysis of zebrafish samples.

In summary, traditional SPE features high enrichment efficiency, effective matrix cleanup, and good compatibility with LC-MS/MS and UPLC-MS/MS, allowing trace analysis of drugs in zebrafish. However, it involves multiple steps (conditioning, loading, washing, elution), consumes relatively large amounts of organic solvents, and is time-consuming, especially for high-throughput sample processing.

#### 3.1.2. Dispersed Solid Phase Extraction

The operational procedures are made simpler by DSPE, which disperses adsorbents straight into zebrafish samples without the use of columns for extraction. DSPE can process zebrafish samples more rapidly and conveniently than standard solid phase extraction [112,113].

Molina Fernandez et al. developed a DSPE for ionic organic compounds that can extract four drugs from zebra embryos at low cost and assay them using GC-MS [59]. Ibuprofen, diclofenac, and naproxen had LODs of 0.4–6.3 ng/mL, RSDs < 8%, and recoveries ranging from 98% to 99% in this investigation. The study demonstrated that test 305 for the Organization for Economic Cooperation and Development might be replaced using this approach. A novel dispersible solid phase extraction technique based on neutral alumina powder acting as a selective adsorber was presented by Shuang Li et al. [114]. Through the optimization of the selective adsorption processes, this study successfully removed the interference of lipid-soluble and hydrophilic chemicals in zebrafish. UPLC-quadrupole orbital trap–high-resolution mass spectrometry (UHPLC-Q-Orbitrap-HRMS) showed a linear range of 0.001–0.02 μg/mL and LODs of 0.01–0.03 mg/kg for patulin metabolites. In conclusion, DSPE is capable of extracting a wide range of substances, such as medicines, insecticides, and chemical compounds. The benefits of DSPE, including its broad variety of applications and ease of use, encourage the advancement of zebrafish determination and analysis techniques.

Overall, DSPE simplifies sample preparation by directly dispersing sorbents into the sample, avoiding cartridges and shortening analysis time. Its versatility enables the extraction of various analytes, including pharmaceuticals and pesticides. However, manual dispersion and separation may lead to poor reproducibility, and additional cleanup is often required to remove sorbents before instrumental analysis.

#### 3.1.3. Magnetic Solid Phase Extraction

MSPE is a modified method of DSPE. It improves the adsorbent in MSPE to a magnetic or magnetizable material, and an external magnetic field is applied to separate the analyte. Therefore, MSPE revolves around the development and application of appropriate magnetic adsorbents.

**Figure 3:**
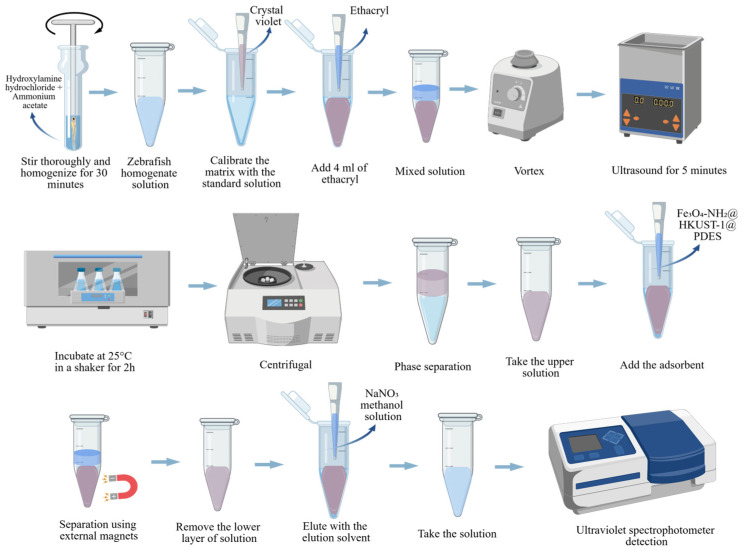
The zebrafish sample pretreatment process was performed using MSPE.

As shown in Figure 3, Weia, Xiaoxiao, and colleagues created poly (deep eutectic solvent)-functionalized magnetic metal–organic framework composites [41,115], which can selectively separate cationic compounds. To pretreat zebrafish samples, they used a composite material that included Fe_3_O_4_, HKUST-1, Fe_3_O_4_-NH_2_, and polymer deep eutectic solvents as adsorbents. The created program demonstrated good linearity in the concentration range of 0.5–10 mg/mL (Malachite Green) and 0.2–5 mg/mL (Crystal Violet). The LODs ranged from 98.19 to 23.97 ng/mL, with RSDs of less than 0.702% [115].

Meanwhile, Youe Zhou et al. created a new type of magnetic nanoparticle of nitrogen-enriched carbon as an SPE material for the investigation of organochlorine pesticides accumulated in zebrafish by GC-MS: dichlorodiphenyltrichloroethane and its metabolites dichlorodiphenyldichloroethylene [116]. In their study, they successfully combined carbon hydrophobicity and nitrogen hydrophilicity to achieve uniform dispersion and quick magnetic separation of N-rich carbon nanoparticles in zebrafish samples. This procedure required only 5 min for analyte adsorption and resolution. Dichlorodiphenyltrichloroethane and dichlorodiphenyldichloroethylene LODs for low ng/mL levels have a relative standard deviation of 6% to 7% [116]. MSPE is a technology used to extract and purify complicated biological matrix samples. It uses a magnetic field to separate samples, resulting in a large mass transfer area and good extraction efficiency.

In conclusion, MSPE integrates the advantages of DSPE with convenient magnetic separation, allowing rapid and efficient isolation of target analytes via an external magnetic field. Its large mass transfer area and simple operation render it highly suitable for complex biological matrices such as zebrafish. However, the synthesis of selective magnetic sorbents is often complicated and costly, and its performance strongly relies on the stability and reusability of magnetic materials.

#### 3.1.4. Solid Phase Microextraction

Although LLE and SPE are widely employed, they still exhibit limitations in processing complex zebrafish sample matrices. These limitations can be quantitatively evaluated using three key performance indicators: enrichment factor (EF), ME suppression, and solvent consumption [56,117].

In terms of the enrichment factor, LLE typically achieves only 5–20-fold enrichment, which is attributed to the restricted volume ratio between the organic solvent and aqueous sample [24]. In comparison, SPE offers slightly higher enrichment efficiency (usually 10–50-fold) but often requires relatively large sample volumes (>100 μL) as well as multiple elution steps [106]. In contrast, advanced SPME coatings, such as those based on metal–organic frameworks (MOFs) or Molecularly Imprinted Polymers (MIPs), have achieved an EF exceeding 150 in zebrafish tissue homogenates, which is significantly superior to that of conventional LLE and SPE [118,119,120]. SPME coatings based on highly graphitized porous carbon/reduced graphene oxide (PC/rGO) can achieve an exceptionally high enrichment factor of up to 126,057 for polycyclic aromatic hydrocarbons, owing to multiple interactions between the coating and target analytes, including hydrophobic interactions, π–π stacking, partitioning effects, and mesopore filling [23]. Recent studies have shown that SPME enables ultrasensitive detection of trace pharmaceutical agents in zebrafish, with limits of detection down to the ng/L or even pg/L level.

Regarding MEs, both LLE and SPE methods tend to co-extract endogenous compounds (e.g., lipids and proteins) from zebrafish homogenates [106]. This co-extraction consequently leads to significant ion suppression (typically 20–35%) during LC-MS/MS analysis, thereby impairing the accuracy and sensitivity of LC-MS/MS quantification. Furthermore, from the perspective of green chemistry, conventional methods consume large volumes of organic solvents (2–10 mL per sample for LLE; 1–5 mL per sample for SPE), resulting in substantial chemical waste that is inconsistent with the principles of sustainable analytical chemistry.

In contrast, SPMEs can reduce ME to below 10% relative to the original level [121,122]. Additionally, its solvent-free nature aligns with the green chemistry principles, and compared to traditional SPE methods (using SPE cartridges), SPME can reduce organic waste generation by more than 90%. These performance advantages underscore the increasing applicability of SPME in drug monitoring in zebrafish.

SPME takes advantage of the different partition coefficients of the target analyte between the sample matrix and the extraction head coating, so as to realize the extraction and enrichment of the sample. Kun Qian et al. created an efficient method for isolating nonylphenols from zebrafish samples using SPME based on two materials: polydimethylsiloxane and divinylbenzene [105]. In their study, SPME was combined with HPLC to quantify nonylphenol in zebrafish samples. The method had a high sensitivity (LOD of nonylphenol 0.4184 µg/L) and a good precision (RSDs of nonylphenol 2.25%).

SPME can realize the integrated operation of sample extraction, enrichment and injection. Yang, Huinan et al. created a new in situ mass spectrometry approach for quick, in situ, microscale measurement of perfluoroalkyl compounds in zebrafish [123]. The established surface-coated probe–nanoESI-MS technique detects 11 chemicals in zebrafish in a single sample run. The LODs of seven perfluoroalkyl substances range from 0.06 to 0.8% and RSDs range from 0.5 to 500% [123]. Three lipids from zebrafish and big fleas were tested to determine the selectivity of the newly designed surface-coated probe–nanoESI-MS technique for analytes. Xue Xiao et al. also created a method for in vivo, in situ, and microscale compound trace analysis in zebrafish (Malachite Green and Crystal Violet) [124]. They designed a new biocompatible surface-coated solid phase microextraction probe based on a tungsten microanatomical probe. After employing this procedure to enrich zebrafish samples, mass spectrometry analysis can be performed directly without the need for any extra sample preparation processes. The developed program showed good linearity over a concentration range of 0.1 to 10 ng/mL (Malachite Green, Crystal Violet, Leuco Malachite Green, Leuco Crystal Violet). The LODs ranged from 0.014 to 0.023 ng/mL, with RSDs of less than 9.1% [124]. Meanwhile, Jiewei Deng et al. used the same probe to accurately detect and identify a wide variety of lipid components in zebrafish [125].

**Figure 4:**
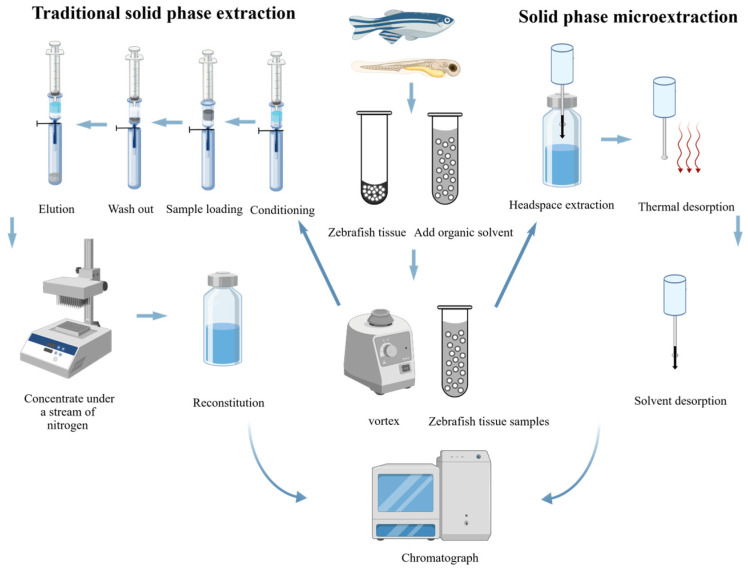
The traditional SPE and SPME processes for sample pretreatment of zebrafish.

As shown in Figure 4, SPME is an effective and adaptable method for the pretreatment of biological materials [41]. The combination of SPME, headspace extraction, and membrane protection extraction may effectively deal with problematic volatile compounds.

SPMEs presents distinct advantages over conventional methods, such as higher enrichment efficiency, weaker ME, and eco-friendliness owing to solvent-free operation. Its integration of sampling, extraction, and injection in one step significantly improves throughput. However, fiber coatings and extraction conditions demand careful optimization, and fiber fragility limits its service life.

### 3.2. Solid Phase Extraction Solvent

#### 3.2.1. Carbon-Based Sorbents

Carbon-based sorbents are commonly employed in zebrafish sample pretreatment due to their strong biocompatibility and adsorption characteristics [126]. N. Molina-Fernandez et al. employed C18 to identify four NSAIDs in zebrafish, with recovery rates ranging from 99% to 101% [59]. The scientists compared the extraction of weakly polar compounds in zebrafish using C18, Florisil, Primary Secondary Amine, and Molecularly Imprinted Polymers [59]. They discovered that C18, a non-polar adsorbent with a long-chain alkyl group, was capable of efficient extraction via hydrophobic interactions. Jon Rose et al. also employed C18 sorbents to assess vitellogenin levels in rainbow trout, fat-headed trout, phoxinus japonicus, and zebrafish [127]. After C18 treatment, zebrafish recovered faster than other species, reaching approximately 85%. Niu Panhong et al. developed a cobalt magnetic polystyrene microsphere-derived carbon (C-Co@PST) for enrichment in zebrafish [104]. The addition of a hydrophobic octadecyl group to the adsorbent increased the adsorption site of the analyte. The C-Co@PST in this paper can be separated by external magnetic field, which is helpful to improve the experimental efficiency. The application of carbon-based sorbents to zebrafish samples requires special attention to lipid removal. The high lipid content in zebrafish whole-body homogenates is much higher than in plasma or urine, which can competitively adsorb onto non-polar sorbents such as C18 and reduce analyte binding. Thus, additional lipid removal steps (e.g., freezing-induced precipitation, hexane washing) are often required during sample preparation. In summary, the carbon-based adsorbent is a non-toxic, easy-to-operate, low-cost and highly extractable adsorbent for zebrafish.

In summary, carbon-based sorbents (e.g., C18) exhibit excellent biocompatibility, high extraction efficiency via hydrophobic interactions, and good compatibility with magnetic separation after functionalization. They are non-toxic, cost-effective, and easy to handle. Nevertheless, their non-specific adsorption may lead to the co-extraction of interferences, and their performance is highly dependent on the polarity of target analytes.

#### 3.2.2. Silan-Based Adsorbent

Silica-based sorbents typically have a large specific surface area and abundant pore structure, which allows them to efficiently adsorb target analytes in biological samples [128]. P. Navarro et al. employed Florisil sorbent to extract nonylphenol, octylphenol, and 17-estradiol from zebrafish tissues [103]. The scientists compared SPE to gel permeation chromatography and discovered that SPE utilizing Florisil adsorbents resulted in cleaner chromatograms. This is because Florisil’s porous nature allows it to more efficiently absorb non-polar or weakly polar contaminants from zebrafish samples [103]. In another study, a variety of silicon-based adsorbents were applied to zebrafish to compare their performance. The silica-based surface in the hydrophilic–lipophilic balance sorbents is modified by specific groups, such as quaternary ammonium salts, which are both hydrophilic and hydrophobic and are able to specifically process biological samples. Mei Yang et al. compared the retention ability of three SPE fillers, Florisil, C18, and hydrophilic–lipophilic balance, to target compounds in zebrafish [99]. Compared to Florisil and C18, they discovered that the application of hydrophilic–lipophilic balance was more adaptable and had a higher retention rate. The 26 target compounds that were examined utilizing hydrophilic–lipophilic balance had recoveries ranging from 70.3% to 118.8% [99,115]. Yaxi Li et al. identified six steroid hormones in zebrafish samples using hydrophilic–lipophilic balance. By integrating SPE with ultra-performance liquid chromatography–time of flight mass spectrometry (UPLC-TOF-MS), this approach significantly cuts the determination time to 6 min [106]. In this paper, 0.1% formic acid was added to the sample solution to improve the extraction efficiency. This is due to the hydrophobic and π–π interactions between the sample and the hydrophilic–lipophilic balance. Although silica-based sorbents function via similar extraction mechanisms across biological matrices, their application to zebrafish samples requires special consideration. Zebrafish matrices exhibit high pigmentation that may remain in extracts and interfere with UV detection, unlike mammalian tissues or plasma. This matrix-specific interference demands careful optimization when using silica-based sorbents for zebrafish analysis.

Silica-based sorbents possess large surface areas and tunable surface modifications (e.g., C18, Florisil), enabling selective extraction of target analytes. Notably, HLB sorbents exhibit dual hydrophilic–lipophilic interactions, improving versatility for various compounds. However, silica-based materials show limited PH stability, and their performance can be impaired by water or polar solvents in the sample matrix.

#### 3.2.3. Polymer and Polymeric Materials

Polymer and polymeric materials are widely used in SPE. They are highly efficient adsorbents for the extraction of analytes in zebrafish due to their wide PH applicability and multiple sites of action. Drugs from zebrafish have been extracted using a polymer called polystyrene. Depending on the extract’s acidity or baseness, researchers frequently employ various mixing patterns. Guowei Wang et al. used a weak anion mixed-mode polystyrene sorbent to determine bis(1,3-dichloro-2-propyl) phosphate and di-n-butyl phosphate in zebrafish [102]. The adsorbent can selectively adsorb negatively charged molecules with a recovery rate of 67.3 to 120.3%. Eva Prats et al. used Ostro™ 96-Well Plate and Oasis^®^ WCX as adsorbents to treat zebrafish samples [129]. These polymers combine ion-exchange and reversed-phase mixing modes into a single material with higher selectivity. The researchers also made a coating of the polymer to cover the fibers for efficient extraction. Kun Qian et al. fabricated polymer coatings by the polymerization of polydimethylsiloxane and divinylbenzene under mild conditions. The coating is applied to the fibers to extract zebrafish samples with high efficiency [105]. Chitosan is a low-cost, biodegradable polymer with excellent biocompatibility. Jiewei Deng et al. developed an octadecyldimethyl [3-(trimethoxysilyl) propyl] ammonium chloride–chitosan composite adsorbent for drug extraction from zebrafish [125].

In conclusion, polymer-based sorbents boast wide PH tolerance, diverse interaction sites (e.g., ion-exchange, reversed-phase), and customizable formats (e.g., fibers, coatings, composites). Their high selectivity and reusability are well-suited for complex zebrafish matrices. However, synthesis is labor-intensive, batch consistency remains an issue, and some polymers may swell/shrink in organic solvents, compromising efficiency.

## 4. Evaluation Strategies for Matrix Effects

Zebrafish samples harbor complex biological matrices abundant in endogenous substances, including lipids, proteins, phospholipids, and salts. The high lipid content in adult zebrafish whole-body samples, the small sample volume from larvae or embryos, and diverse experimental settings further aggravate matrix interference, hindering method standardization and inter-study comparability. Therefore, systematic evaluation and mitigation of MEs are essential for reliable quantification.

### 4.1. Assessment of MEs

#### 4.1.1. Post-Extraction Addition Method

**Figure 5:**
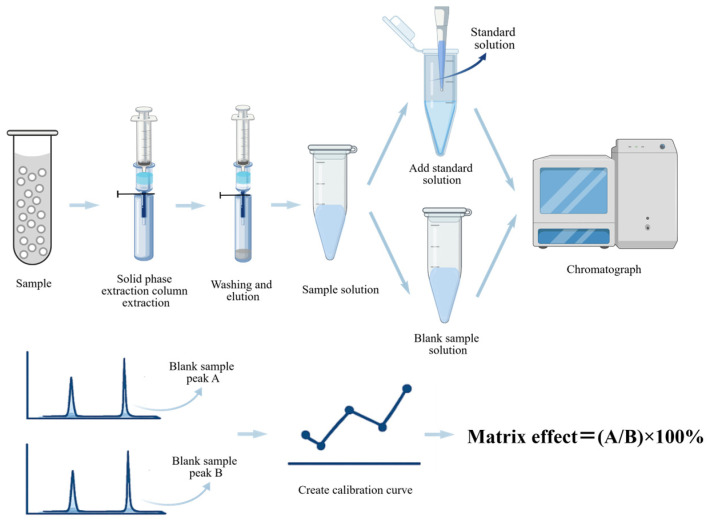
The process of determining zebrafish samples using the post-extraction addition method.

The post-extraction addition method serves as the standard procedure for evaluating and quantifying MEs in mass spectrometry-based analysis [130]. As shown in Figure 5, it compares the response of analytes spiked into blank matrix after extraction with that of neat standards at identical concentrations [41]. ME is calculated as follows:

ME (%) = (Peak area in post-extraction spiked matrix/Peak area in neat solution) × 100%

An ME value of 100% indicates no matrix interference, whereas values above or below 100% represent ion enhancement or suppression, respectively. According to the Food and Drug Administration and European Medicines Agency guidelines, MEs should be assessed using at least six batches of blank matrix, with a coefficient of variation ≤ 15% for the internal standard-normalized matrix factor [131,132].

A major merit of this approach is that it distinguishes MEs from extraction recovery, as signal variations arise exclusively from ionization processes rather than analyte loss during sample preparation. This facilitates targeted optimization of either cleanup procedures or extraction efficiency. For instance, Li Ya et al. applied this strategy during the development of a UPLC-TOF-MS method for steroid hormones in zebrafish whole-body homogenates and observed notable matrix-induced ion interference, highlighting the necessity of effective compensation strategies for such complex matrices [106].

Nevertheless, the requirement of a blank matrix represents a key limitation, as it is often challenging to acquire for certain zebrafish tissues. Therefore, while indispensable for method validation, the post-extraction addition method should be coupled with appropriate calibration strategies to ensure reliable quantification in routine analysis.

#### 4.1.2. Tissue-Specific Assessment Strategies

When analyzing drug or contaminant distribution in various zebrafish organs (e.g., brain, liver, muscle), MEs can differ significantly across tissues. To address this issue, Luan et al. established a Tissue-Specific Ionization Efficiency Factor (TSF) strategy [47]. By evaluating MEs in individual tissues (e.g., gill, egg, brain) and applying corresponding correction factors, this approach minimizes matrix interference and improves quantitative accuracy. Using TSF, they demonstrated that MEs in six zebrafish regions decreased in the following order: egg > viscera > gill > ventral fin > brain > muscle [47].

The main advantage of the TSF strategy is its high tissue specificity and in situ calibration capacity. Nevertheless, its application is limited by several drawbacks. First, the determination of correction factors is labor-intensive, rendering it unsuitable for high-throughput analysis. Second, accurate ME evaluation remains challenging for tiny or poorly defined tissues (e.g., specific brain nuclei). Therefore, this method is more appropriate for spatial distribution studies than for routine batch analysis.

### 4.2. Compensation Strategies for MEs

#### 4.2.1. Matrix-Matched Calibration

Matrix-matched calibration is one of the most common and direct strategies for compensating MEs. By preparing calibration standards in a blank matrix that mirrors the composition of real samples, this approach ensures that standards undergo identical matrix interference, thereby automatically correcting signal bias during quantification [133].

For zebrafish whole-body homogenates, a blank matrix obtained from analyte-free individuals can be processed and used to prepare calibration standards. Gwachha et al. employed matrix-matched calibration for LC-MS/MS quantification of methamphetamine in 5-day-old zebrafish larvae, achieving recoveries of 92–120% [48]. Kislyuk et al. constructed linear calibration curves using matrix-matched standards by spiking the target compounds into evaporated blank zebrafish matrix samples at a minimum of three concentration levels [79]. In parallel, linear calibration curves were also prepared using standard solutions in 94:6 (*v*/*v*) H_2_O:ACN. MEs were corrected by comparing the slopes of the calibration curves obtained from matrix-matched and neat standard solutions. Following this correction, the MEs for eight compounds, including bupropion, ranged from 81% to 103% [79]. These studies confirm that matrix-matched calibration is applicable to various zebrafish tissue types and analytes with diverse physicochemical properties.

Despite its widespread application, matrix-matched calibration has inherent advantages and limitations. Its primary advantage is simplicity and cost-effectiveness, as it only requires blank matrix and unlabeled standards—making it particularly useful when isotopically labeled analogs are unavailable. However, this approach has notable limitations: Acquiring sufficient blank matrices is challenging for micro-samples (e.g., zebrafish brain or liver), and it fails to correct for analyte loss during sample preparation.

#### 4.2.2. Isotope Internal Standard Method

Isotopically labeled internal standards represent a gold-standard strategy for compensating MEs. Stable isotope-labeled analogs are added to each sample prior to processing. Since they share nearly identical chemical properties and chromatographic behavior with the target analytes, yet can be distinguished by mass spectrometry, quantification using the analyte-to-internal standard peak area ratio effectively corrects for both analyte loss during sample preparation and ionization fluctuations caused by matrix interference.

This approach has been widely adopted in zebrafish studies [129,134]. Li et al. used cortisol-d4 as an internal standard (50 ng/mL) for the quantification of steroid hormones in zebrafish whole-body homogenates [106]. After confirming MEs by the post-extraction addition method, the internal standard was introduced before sample preparation, efficiently compensating for signal fluctuations and ensuring accurate quantification of trace hormones in complex biological matrices. Beyond conventional small-molecule analysis, isotopically labeled internal standards also play an indispensable role in quantifying extremely low-abundance endogenous proteins. Using a bacterial-based stable isotope labeling (SILIB) strategy coupled with UHPLC-MRM/MS, researchers employed fully labeled [^13^C,^15^N]-zFXN-M as an internal standard and successfully quantified zFXN-M in zebrafish embryos (120.9 ± 20.1 amol per embryo) and adult whole-body homogenates (2.26 ± 0.44 ng/mg protein) [135]. This method effectively compensated for matrix interference in complex biological samples and has become a key technique for validating gene-edited zebrafish models and quantifying ultra-trace endogenous proteins.

The isotopically labeled internal standard method enables simultaneous correction of both MEs and extraction losses. Since the internal standard undergoes identical sample preparation and ionization processes as the native analyte, this approach effectively compensates for interferences arising from both stages. This dual correction capability is particularly valuable for complex matrices such as zebrafish tissues, where ion suppression and variable extraction recovery are common challenges. However, this method is limited by the restricted availability and high cost of isotopically labeled standards, especially for emerging or novel compounds.

## 5. Future Perspectives in Zebrafish Sample Pretreatment

In recent years, researchers have developed a variety of efficient sample pretreatment techniques for drug analysis in zebrafish models. This is particularly the case with respect to extraction forms, extraction media, and extraction assistive techniques. These techniques are designed to reduce matrix interference and to enrich analytes, thereby improving assay efficiency.

At present, the pretreatment technology of zebrafish samples in laboratory analysis is relatively mature, but the pretreatment technology for field rapid detection still needs to be further developed and promoted. In addition, as the concept of green chemistry and sustainable development has gained prominence, researchers also need to explore more environmentally friendly and efficient sample pretreatment materials and methods to promote the wide application of zebrafish models in drug safety evaluation.

In the future, the focus of zebrafish sample pretreatment technology will remain on the development of extraction media with a high recovery rate, high sensitivity, and high anti-interference capability, as well as the continuous improvement of auxiliary extraction technologies. The development of green extraction media with high affinity and efficient elution characteristics, along with the design of new pretreatment methods suitable for different experimental systems, will be key to achieving high extraction efficiency. These advancements will greatly simplify the operation process, accelerate analysis speed, and promote the development of zebrafish sample pretreatment toward standardization and automation, ultimately providing more convenient, reliable, and efficient solutions for life science research and application.

## 6. Conclusions

Zebrafish have emerged as a valuable vertebrate model for drug discovery and safety assessment, which demands robust and reliable sample pretreatment strategies. This review summarizes recent advances in extraction techniques and sorbent materials, with a critical discussion on the advantages and limitations of each approach. Although considerable progress has been achieved in enhancing enrichment efficiency, alleviating MEs, and reducing organic solvent consumption, challenges still exist in method standardization, practical applicability, and the integration of green chemistry principles. Future research should focus on the development of intelligent, eco-friendly, and automated pretreatment systems to fully exploit the potential of zebrafish models in pharmaceutical analysis and drug development.

## Figures and Tables

**Table 1:** Conventional liquid–liquid extraction techniques for the determination of drugs in zebrafish.

Analyte	Extractant	Amount of Solvent (μL)	Extraction Time (min)	Method of Analysis	LODs	RSDs (%)	Linearity Range	Recovery (%)	Ref.
3-methoxytyramine dopamine normetanephrine norepinephrine epinephrine serotonin 3-methoxytyramine	methanol	350	40	CC-MS ^1^	0.4% 0.7% 0.4% 0.8% 28e% 0.5%	7.4 6.8 9.3 2.5 5.6e 4.3	1–100% 1–100% 1–100% 1–100% 10–100% 1–100%	98 96	[43]
bonded long-chain fatty acids	ethyl acetate	450	0.67	GC-μECD ^2^	12 ng/g	6.3	0.40–55 μg/g	97 ± 6	[44]
cefathiamidine	Triton X-114/ NaCl/ formic acid	1110	8	HPLC	0.038 μg/g	0.5–4.9	0.15–489 μg/g	97.6–109.7	[45]
metformin	methanol	450	30	CE-MS	4 μg/L	6	-	90	[46]
perfluorooctanoic acid perfluorooctane sulfonate	methanol/H_2_O/ ammonium acetate	1	0.067	LESA-MSI ^3^	6.7 pg/L 22.2 pg/L	9.5 8.0	0.1–50 μg/L 0.1–50 μg/L	-	[47]
methamphetamine	acetonitrile	100	6	LC-MS/MS ^4^	-	7.9	-	92–120	[48]
fipronil	acetonitrile	8000	60	HPLC	0.025 mg/kg	<5.27	0.025–1 mg/kg	92–112	[49]
polycyclic aromatic hydrocarbons	dichloromethane	-	10	GC-MS	0.1–1 ng/g	<20	-	70–120	[50]
polycyclic aromatic hydrocarbons	n-hexan/ dichloromethane	10,000	1500	GC-MS/MS ^5^	-	-	5–1000 μg/L	82.7 (Phenanthrene) 80.8 (Anthracene) 94.3 (Fluoranthene) 91.4 (Pyrene)	[51]
pentachlorobiphenyl	n-hexane	-	-	GC-MS	1 mg/kg	<11.9	-	91.3–100	[52]
tebuconazole	acetonitrile/ tert-butyl methyl ether/ ethyl acetate	2000	30	HPLC-HRMS	0.01 ng/mL	10.4	0.01–200 ng/mL	1.2–49.4	[53]
rac-glufosinate- ammonium glufosinate-PNAG 3-methyl phosphonic N-acetyl glutamate 2-methyl phosphinico- acetic acid	methanol- water dichloromethane	15,000 20,000	1020	HPLC-HRMS	0.002 mg/kg 0.002 mg/kg 0.004 mg/kg 0.006 mg/kg 0.006 mg/kg	<10.03	0.002–0.2 μg/mL	77–104	[54]
tributyl phosphine bifenox aniline yellow	acetonitrile water	70 70	3	GC-MS/MS	1 ng/mL 1 ng/mL 5 ng/mL	77.8 123 59.0	1–300 ng/mL	-	[55]
oxysterols	CHCl_3_ methanol	300 300	90	HPLC-MS/MS ^6^	22–65 pg/100 embryos	≤19	0.5–250 ng/mL	25–50	[56]
Xinjunan	methanol n-hexane sodium- hydroxide concentrated-ammonia water	5000 15,000 8000 2000	3	LC-MS/MS	5 μg/kg	2.21–3.81	0.1–40 mg/kg	102.1–105.2	[57]
polybrominated diphenyl ethers methoxyl polybrominated diphenyl ethers	n-hexane dichloromethane	55,000 30,000	15	GC-MS/MS	0.092–17.0 ng/g	<21.5	4.0–500 ng/mL	71.6–133	[58]

^1^ CE-MS (capillary electrophoresis–mass spectrometry), ^2^ GC-μECD (gas chromatography–micro electron capture detector), ^3^ LESA-MSI (analysis liquid extraction surface analysis–mass spectrometry imaging), ^4^ LC-MS/MS (liquid chromatography–tandem mass spectrometry), ^5^ GC-MS/MS (gas chromatography–tandem mass spectrometry), ^6^ HPLC-HRMS (high-performance liquid chromatography–high-resolution mass spectrometry).

**Table 2:** Summary of liquid phase extraction techniques and solvents for zebrafish analysis.

Extraction Technique	Typical Solvents	Time of Processing (Including Waiting/ Reaction/Centrifugation)	Recommended Analyte Classes	Key Advantages	Limitations
Traditional LLE	Ethanol, methanol, chloroform, hexane	10–30 min/batch	Broad range (polar to non-polar)	Simple, cost-effective, room temperature operation	High solvent consumption, low enrichment, emulsion formation
ASE	Acetone/hexane, dichloromethane	20–40 min/batch	Non-polar to medium-polar compounds (PCBs, PAHs)	High efficiency, automated, low solvent use	Requires specialized equipment, risk of thermal degradation
HF-LPME	Toluene, 1-octanol	40–80 min/batch	Medium to non-polar (two-phase); ionizable (three-phase)	High enrichment (up to 279-fold), minimal solvent use, excellent cleanup	Long extraction time, manual handling, fiber fragility
DLLME	Hexane, chlorinated solvents	5–15 min/batch	Broad range, especially hydrophobic compounds	Rapid, high enrichment, simple operation	Requires dispersion optimization, solvent toxicity concerns
Ultrasound-assisted DLLME	Hexane, acetonitrile	10–20 min/batch	Heat-sensitive compounds	Non-thermal, preserves biological activity	Longer sonication time (2 h in some cases)

**Table 3:** Solid phase extraction technique for the determination of drugs in zebrafish.

Analyte	Sorbent	Amount of Sorbent (mg)	Sorption Time (min)	Eluent	LODs	RSDs (%)	Linearity Range	Recovery (%)	Ref.
Pregnane Testosterone	C18	-	-	methanol/ water solution (30:70, *v*/*v*)	0.32 ng/g 0.01 ng/mL	7.9 4.6	1.0–100 ng/mL 1.0–100 ng/mL	74.4 86.0	[99]
Triptolide	Oasis™HLB solid phase extraction cartridge	-	-	methanol	0.02 ng/mL	6.79–7.88	0.115–360 ng/mL	87.6–105.3	[100]
Triphenyl phosphate Tetrabromobisphenol A Ethylhexyl diphenyl phosphate Hexabromocyclododecane	Bond Elut ENV sorbent	500	5	ethyl acetate	0.17 μg/g 0.05 μg/g 0.04 μg/g 0.10 μg/g	6.6 7.4 5.2 6.8	0.52–20 μg/g 0.15–20 μg/g 0.12–20 μg/g 0.31–50 μg/g	98 84 58 82	[101]
Bis(1,3-dichloro-2-propyl) phosphate Di-n-butyl phosphate	Weak anion exchange SPE cartridge	500	10	methanol	0.9 ng/g 0.4 ng/g	-	-	83.2–120.3 67.3–95.9	[102]
Nonylphenols Octylphenols 17β-Estradiol	Florisil^®^	500 10,000	-	ethyl acetate	827 ng 0.04 ng 1.5 ng	10–31	-	106–126 106–126 106–126	[103]
Bisphenol A	-	20	10	methanol and acetonitrile mixture (1:1, *v*/*v*)	0.033–0.110 μg/L	2.1–7.5	0.2–200 μg/L	84.13–98.57	[104]
4-nonylphenol	Polydimethylsiloxane/divinylbenzene coated fiber	-	20	-	0.4184 µg/L	2.25	20.92–2.092 × 10^5^ µg/L	77.797–89.274	[105]
Cortisol Testosterone Androstenedione 11-Deoxycortisol 11-Deoxycorticosterone 17-Hydroxyprogesterone	-	100	-	75% methanol/H_2_O containing 2% acetic acid	0.1% 0.1% 0.1% 0.2% 0.2%	6.6 2.8 3.1 5.84	0.3–200 0.3–200 0.3–200 0.7–200 0.7–200	97.6 104.9 107.9 93.1 90.6	[106]
2,2′,4,4′-Tetrabromodiphenyl ether Methoxylated polybrominated diphenyl ethers Hydroxylated polybrominated diphenyl ethers	Florisil	50	4	n-hexane methyl tert-butyl -ether (1:1, *v*/*v*)	0.1–0.3 µg/L	<12	-	-	[107]
Ibuprofen Naproxen Diclofenac sodium Clofibric acid	C18 sorbent	100	10	acetonitrile	0.4–6.3 ng/mL 0.4–6.3 ng/mL 0.4–6.3 ng/mL 0.1–1.9 ng/mL	<8	5–200 ng/mL 5–200 ng/mL 5–200 ng/mL 50–1500 ng/mL	99 ± 2 99 ± 2 99 ± 2 99 ± 2	[59]

**Table 4:** Summary of solid phase extraction techniques and sorbents for zebrafish analysis.

Extraction Technique	Typical Sorbents	Recommended Applications	Time of Processing (Including Waiting/ Reaction/Centrifugation)	Key Advantages	Limitations
Traditional SPE	C18, HLB, Florisil	Trace analysis, multi-class drugs	40–90 min/batch	High enrichment, excellent LC-MS compatibility	Multiple steps, solvent-intensive, time-consuming
DSPE	C18, PSA, alumina	Rapid screening, pesticides, NSAIDs	20–30 min/batch	Simple, fast, no cartridges	Manual variability, sorbent removal needed
MSPE	Fe_3_O_4_-based composites, C-Co@PST	Small-volume samples (larvae/embryos), rapid isolation	10–20 min/batch	Fast magnetic separation, large mass transfer area	Complex sorbent synthesis, cost considerations
SPME	PDMS, DVB, MOFs, MIPs, PC/rGO	Trace analysis, in vivo sampling, volatiles	30–70 min/batch	Solvent-free, integrates extraction/injection, high EF (up to 126,057)	Fiber fragile, coating optimization required

## Data Availability

No new data were created or analyzed in this study. Data sharing is not applicable.

## Abbreviations

The following abbreviations are used in this manuscript:

LPE	Liquid phase extraction
SPE	Solid phase extraction
HPLC	High-performance liquid chromatography
LC-MS	Liquid chromatography–mass spectrometry
LLE	Liquid–liquid extraction
SPME	Solid phase microextraction
LPME	Liquid phase microextraction
LC	Liquid chromatography
SFLLE	Supercritical fluid liquid–liquid extraction
ASE	Accelerated solvent extraction
HF-LLME	Hollow fiber liquid–liquid microextraction
DLLME	Dispersive liquid–liquid microextraction
LODs	Limit of detection
RSDs	Relative standard deviation
UAMC	Ultrasound-assisted micellar cleanup
UHPLC-MS/MS	Ultra-high-performance liquid chromatography–tandem mass spectrometry
GC-MS	Gas chromatography–mass spectrometry
LOQs	Limit of quantitations
GC-μECD	Gas chromatography–micro electron capture detector
CE-MS	Capillary electrophoresis–mass spectrometry
LESA-MSI	Analysis liquid extraction surface analysis–mass spectrometry imaging
LC-MS/MS	Liquid chromatography–tandem mass spectrometry
GC-MS/MS	Gas chromatography–tandem mass spectrometry
HPLC-HRMS	High-performance liquid chromatography–high-resolution mass spectrometry
PAHs	Polycyclic aromatic hydrocarbons
DSPE	Dispersive solid phase extraction
MSPE	Magnetic solid phase extraction
LC–MS/MS	Liquid chromatography–tandem mass spectrometry
UPLC-MS/MS	Ultra-performance liquid chromatography–tandem mass spectrometry
UPLC-HRMS	Ultra-high-performance liquid chromatography–high-resolution mass spectrometry
UHPLC-Q-Orbitrap-HRMS	Ultra-performance liquid chromatography–quadrupole orbital trap–high-resolution mass spectrometry
C-Co@PST	Cobalt magnetic polystyrene microsphere-derived carbon
UPLC-TOF-MS	Ultra-performance liquid chromatography–time of flight mass spectrometry
ESI-MS	Electrospray ionization mass spectrometry
MEs	Matrix effects
